# Pro-Tumorigenic Effect of Continuous Cromolyn Treatment in Bladder Cancer

**DOI:** 10.3390/ijms26041619

**Published:** 2025-02-14

**Authors:** Lucija Franković, Marina Degoricija, Ivana Gabela, Katarina Vilović, Jelena Korac-Prlic

**Affiliations:** 1 Laboratory for Cancer Research, Department of Immunology and Medical Genetics, School of Medicine, University of Split, 21000 Split, Croatia; lucija.frankovic@mefst.hr (L.F.);; 2 Department of Medical Chemistry and Biochemistry, School of Medicine, University of Split, 21000 Split, Croatia; 3 Department of Pathology, Forensic Medicine and Cytology, University Hospital of Split, 21000 Split, Croatia; 4 Department of Anatomy, School of Medicine, University of Split, 21000 Split, Croatia

**Keywords:** cromolyn, bladder cancer, MB49 cancer cells, mast cells

## Abstract

Globally, bladder cancer is the sixth most frequently diagnosed cancer among men. Despite the increasing availability of immunomodulatory treatments for bladder cancer, the survival rates are still low, which calls for potential new drug-repurposing targets. This study aimed to investigate the effects of cromolyn, a mast cell (MC) stabilizer in allergic reactions, on a subcutaneous tumor model with a syngeneic mouse MB49 bladder cancer cell line. A concentration of 50 mg/kg of cromolyn was daily administered intraperitoneally in a 4-day therapeutic protocol to mice with established tumors and in a continuous 11-day protocol which started one day prior to the subcutaneous injection of tumor cells. Therapeutic treatment demonstrated a marked downregulation of genes related to angiogenesis and upregulation of genes related to cytotoxic T-cell and NK cell activity. Conversely, continuous cromolyn treatment suppressed genes involved in immune cell recruitment and activation, as well as apoptotic and necroptotic pathways, leading to a greater tumor burden (+142.4 mg [95CI + 28.42, +256.4], *p* = 0.0158). The same pro-tumorigenic effect was found in mast cell-deficient mice (Kit^W-sh/W-sh^ + 301.7 mg [95CI + 87.99, 515.4], *p* = 0.0079; Cpa3^Cre/+^ +107.2 mg [95CI − 39.37, +253.57], *p* = 0.1423), indicating that continuous cromolyn treatment mostly acts through the inhibition of mast cell degranulation. In summary, our results demonstrate the distinct effects of cromolyn on tumor progression, which depend on the protocol of cromolyn administration.

## 1. Introduction

Globally, bladder cancer is the sixth most frequently diagnosed cancer among men, while in Europe, it ranks fourth. In the year 2022 alone, there were over half a million new cases reported, resulting in more than 220,000 deaths [1]. In addition to high prevalence, bladder cancer is also linked with significant morbidity, mortality, and economic burden. Around 75% of newly diagnosed cases present as superficial urothelial carcinoma; however, high-grade, recurrent non-muscle invasive bladder cancer (NMIBC) poses a substantial risk of progressing to invasion of muscle and metastasis. For NMIBC, the conventional approach involves administering intravesical Bacillus Calmette–Guérin (BCG) therapy following the initial transurethral resection of the bladder tumor. Patients with muscle-invasive bladder cancer (MIBC) typically undergo cystectomy, with or without chemotherapy [2]. In recent years, immunotherapy using checkpoint inhibitors has emerged as a promising treatment for cancers. The US Food and Drug Administration has approved five drugs that target either PD-1 or PD-L1 for treating metastatic urothelial carcinoma. Systemic immunotherapy employing immune checkpoint blockade (ICB) has shown notable effectiveness in around 15–25% of patients with metastatic disease [3].

Given the low overall survival rates for cancer patients and the high costs associated with developing new anti-cancer drugs, there is a growing need to explore combining conventional therapies with new approaches including drug repurposing to reduce tumor growth, metastatic potential, and drug resistance [4]. The use of immunotherapy in cancer treatment highlights the immune system’s powerful role in modulating tumor growth and progression. Consequently, medications that modulate the immune system hold significant potential in combination with conventional cancer treatment protocols. Immune cells are a crucial component of the complex and dynamic tumor microenvironment. However, cancer cells can manipulate this microenvironment to their advantage, facilitating immune evasion, tumor growth, and metastasis [5]. Among immune cells, MCs are tissue-resident cells derived from the myeloid lineage [6]. Within tumors, MCs can adopt two distinct phenotypes, either a pro-inflammatory phenotype that supports anti-tumor activity and an immunosuppressive phenotype that promotes tumor progression. This duality explains the inconsistent prognostic value of MCs, as studies often report conflicting findings. Moreover, the role of mast cells in the development and progression of bladder cancer remains unclear [7,8,9]. Additionally, evidence suggests that MCs may interfere with anti-PD-1 therapy in mice, reducing its effectiveness [10].

Cromolyn, a mast cell stabilizer, is commonly prescribed for treating asthma and allergy symptoms [11]. The mechanism of cromolyn action is based on the inhibition of mast cell degranulation by blocking calcium influx into mast cells, thereby preventing the release of prostaglandins, leukotrienes, histamine, and other inflammatory substances [12]. Its effects have been explored in vitro on different cancer cell lines, where it has been shown to reduce cancer cell viability, inhibit proliferation, and induce apoptosis [13]. In colon cancer cell studies, cromolyn demonstrated greater selectivity than the chemotherapeutic agent doxorubicin, inducing apoptosis in cancer cells while sparing healthy ones. Subcutaneous mouse models of colon cancer also suggested that cromolyn could reduce tumor weight, although these differences were not statistically significant compared to doxorubicin, which significantly reduced tumor burden [14].

This study aimed to investigate the effects of cromolyn on bladder cancer using a subcutaneous tumor model with MB49 mouse bladder cancer cells. While cromolyn had no direct effect on bladder cancer cells in vitro, it strongly affected subcutaneous tumors. Strikingly, the protocol of cromolyn administration resulted in contrasting effects, displaying both protumorigenic and antitumorigenic effects.

## 2. Results

### 2.1. Effect of Continuous and Therapeutic Cromolyn Treatment on Subcutaneous Tumors from MB49 Bladder Cancer Cell Line

**Figure 1:**
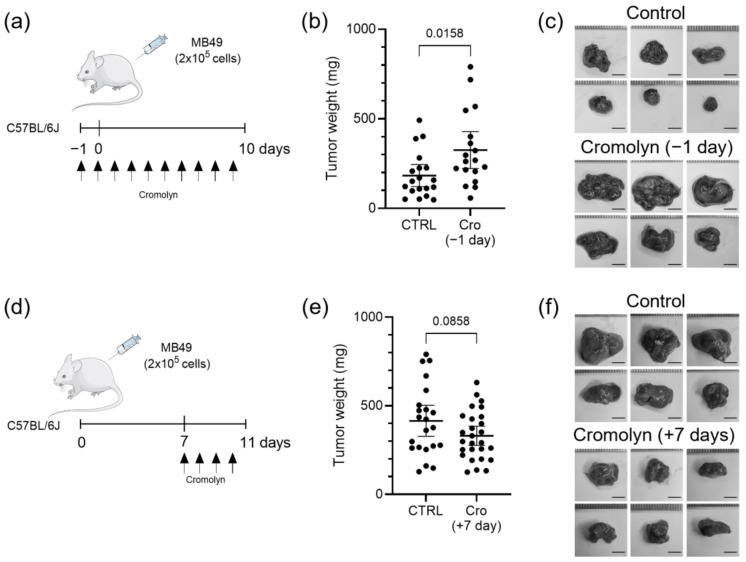
Effect of continuous and therapeutic cromolyn treatment on subcutaneous tumors generated from MB49 murine bladder cancer cell line. (**a**) Schematic representation of continuous cromolyn treatment in mice with subcutaneous tumors from MB49 cell line. (**b**) Tumor weight difference in the control and the cromolyn-treated group at the end of the continuous cromolyn experiment is +142.4 mg [95CI + 28.42, +256.4]. *n* = 19 and 18; Student’s *t*-test, *p* = 0.0158. (**c**) Representative images of subcutaneous tumors at the end of the experiment with continuous cromolyn treatment. Scale bar is 5 mm. (**d**) Schematic representation of therapeutic cromolyn treatment in subcutaneous tumor-bearing mice. (**e**) Tumor weight differences in control and the cromolyn-treated group at the end of the therapeutic cromolyn treatment is −84.46 mg [95Cl − 181.3, +12.37]. *n* = 22 and 27; Student’s *t*-test, *p* = 0.0858. (**f**) Representative images of subcutaneous tumors at the end of the experiment with therapeutic cromolyn treatment. Scale bar is 5 mm. Data are presented as the mean with 95% CI.

To investigate the effects of continuous and therapeutic cromolyn treatment on bladder cancer, we used a subcutaneous mouse model based on the common syngeneic murine MB49 bladder cancer cell line. For continuous treatment protocol, mice were first intraperitoneally injected with cromolyn, followed by subcutaneous injection of MB49 bladder cancer cells the next day. Cromolyn treatment was administered daily until the end of the experiment (Figure 1a). At the end of the experiment, tumors in the cromolyn-treated group were heavier than tumors in the control group with mean difference of +142.4 mg [95CI + 28.42, +256.4], *p* = 0.0158 (Figure 1b,c). In the second therapeutic protocol experiment, mice were first injected with MB49 cells, and cromolyn treatment was administered on day 7 after tumors were established, continuing daily cromolyn administration until the end of the experiment (Figure 1d). We observed an inconclusive effect of cromolyn in the therapeutic treatment group with differences of −84.46 mg [95Cl − 181.3, +12.37], *p* = 0.0858 in mean tumor weights between control and therapeutic cromolyn-treated tumors (Figure 1e,f).

### 2.2. Effect of Continuous Cromolyn Treatment on RNA Expression in Subcutaneous MB49 Bladder Tumors

**Figure 2:**
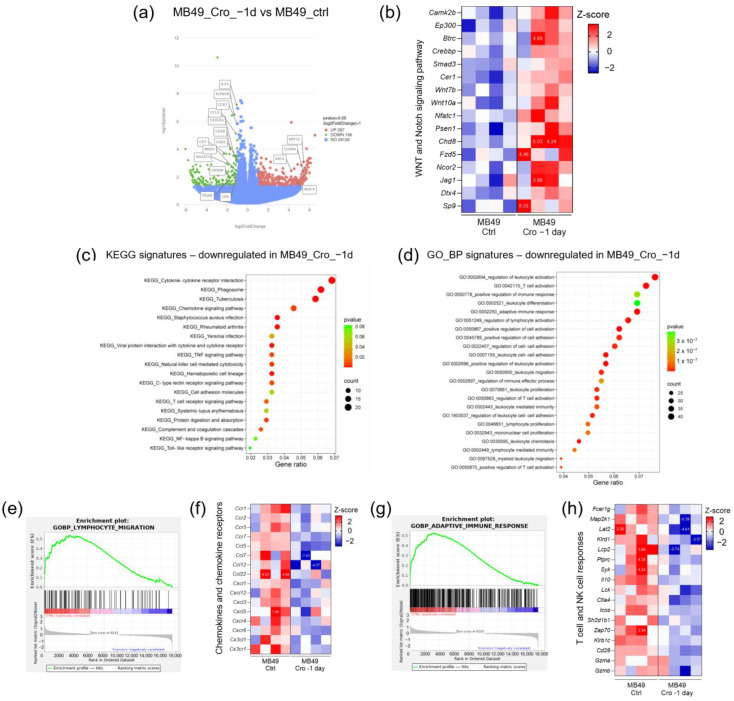
RNA sequencing analysis of subcutaneous tumors from continuous cromolyn-treated mice. (**a**) A volcano plot illustrating the differential gene expression in subcutaneous tumors from mice subjected to continuous cromolyn treatment compared to control mice. (**b**) Heatmap displaying the differentially expressed genes associated with the Wnt and Notch signaling pathway in tumors from continuous cromolyn treatment and control subcutaneous tumors. (**c**) The KEGG pathway enrichment analysis presented as a bubble plot, showing downregulated pathways in subcutaneous tumors following continuous cromolyn treatment compared to the control. (**d**) The GO biological processes enrichment analysis is shown in a bubble plot, highlighting the biological processes downregulated in tumors from continuous cromolyn treatment compared to control tumors. (**e**) The enrichment plot illustrating the expression of genes involved in the lymphocyte migration pathway. (**f**) Heatmap displaying differentially expressed chemokines and chemokine receptors following continuous cromolyn treatment compared to controls. (**g**) Enrichment plot showing the expression of genes associated with the adaptive immune response pathway. (**h**) Heatmap showing differentially expressed genes involved in T-cell and NK cell responses. Numbers indicated on heatmaps are Z scores out of the legend range.

An RNA sequencing analysis of subcutaneous tumors identified 287 upregulated and 196 downregulated genes in tumors from mice treated continuously with cromolyn above the threshold of fold change greater than two and *p*-value < 0.05. Upregulated genes included cytokeratins *KRT4* and *KRT15*, as well as *CLDN4* and *MUC4*, while downregulated genes were predominantly involved in immune signaling pathways (Figure 2a). Notably, genes associated with the Wnt and Notch signaling pathways were upregulated in cromolyn-treated mice (Figure 2b). The KEGG pathway enrichment analysis further highlighted a downregulation of immune response pathways (Figure 2c). The GO biological process analysis confirmed these findings by revealing a reduced expression of genes associated with immune responses in continuous cromolyn-treated mice (Figure 2d), including the downregulation of genes involved in the lymphocyte migration pathway (Figure 2e). Further investigation revealed that continuous cromolyn treatment suppressed specific chemokines and chemokine receptors critical for attracting immune cells (Figure 2f). This collectively contributed to a diminished adaptive immune response, as evidenced by the downregulation of genes in the GO_BP adaptive immune response pathway (Figure 2g). Additionally, we observed a reduced expression of genes associated with cytotoxic T-cell and NK cell responses (Figure 2h).

### 2.3. Decrease in Apoptosis and Necroptosis During Continuous Cromolyn Treatment

**Figure 3:**
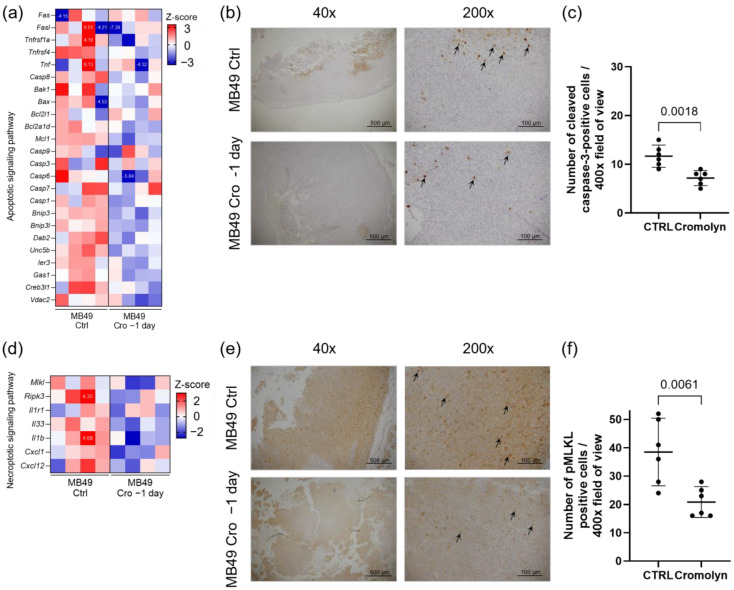
Decrease in apoptosis and necroptosis upon continuous cromolyn treatment. (**a**) Heatmap showing the differentially expressed apoptosis-related genes in tumors from continuous cromolyn-treated mice compared to controls. (**b**) Representative images of immunohistochemical staining of subcutaneous tumor sections, showing cleaved caspase-3-positive cells in the control and continuous cromolyn-treated mice. Arrows indicate cleaved caspase-3-positive cells. Scale bar for 40× and 200× is 500 and 100 μm, respectively. (**c**) The quantification of cleaved caspase-3-positive cells in immunohistochemically stained subcutaneous tumor sections. *n* = 6; Student’s *t*-test, *p* = 0.0018. (**d**) Heatmap showing the differentially expressed necroptosis-related genes in tumors from continuous cromolyn-treated mice compared to control subcutaneous tumors. (**e**) Representative images of immunohistochemical staining of subcutaneous tumor sections, highlighting pMLKL^+^ cells in control and continuous cromolyn-treated mice. Arrows indicate pMLKL^+^ cells. Scale bar for 40× and 200× is 500 and 100 μm, respectively. (**f**) Graphical representation of the quantification of pMLKL^+^ cells in immunohistochemically stained subcutaneous tumor sections. *n* = 6; Student’s *t*-test, *p* = 0.0061. Data are presented as mean with 95% CI. Numbers indicated on heatmaps are Z scores out of the legend range.

Following the observed reduction in the expression of genes related to cytotoxic T-cell and NK cell responses, we additionally analyzed RNA sequencing data for apoptosis-related genes which were also downregulated (Figure 3a). Immunohistochemistry confirmed this result, revealing a marked decrease in cleaved caspase-3-positive cells, a marker of activated apoptosis, in mice treated continuously with cromolyn (*p* = 0.0018) (Figure 3b,c).

Additionally, this group of mice exhibited the downregulation of genes which are essential for the necroptosis pathway, such as *Ripk3* and *MLKL* (Figure 3d). Using immunohistochemistry, we further validated these findings. With an anti-pMLKL antibody, we demonstrated a statistically significant decrease in pMLKL^+^ cells in mice treated continuously with cromolyn (*p* = 0.0061) (Figure 3e,f).

### 2.4. No Direct Effect of Cromolyn Treatment on Bladder Cancer Cell Lines

**Figure 4:**
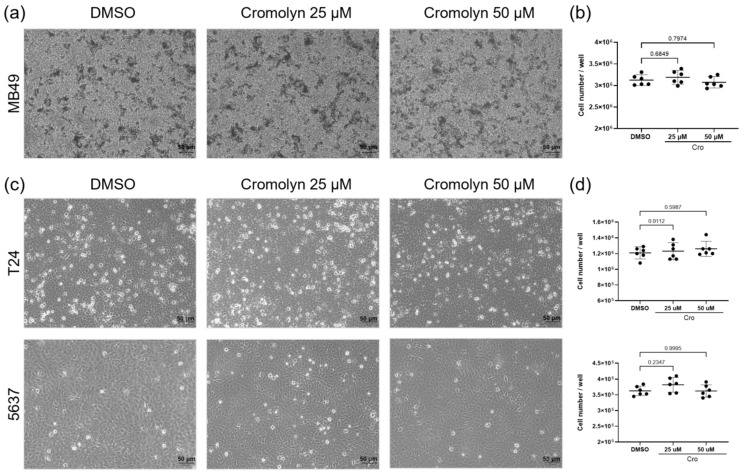
No direct effect of cromolyn treatment on bladder cancer cell lines. (**a**) Representative images of MB49 bladder cancer cells treated with different concentrations of cromolyn (25 μM and 50 μM). Scale bar is 50 μm. (**b**) The quantification of MB49 cells following cromolyn treatment. One-way ANOVA followed by post hoc Tukey test; *p*-value indicated on the graph. (**c**) Representative images of T24 (upper panel) and 5637 (lower panel) bladder cancer cell lines treated with different concentrations of cromolyn. Scale bar is 50 μm. (**d**) The quantification of T24 (upper panel) and 5637 cells (lower panel) after cromolyn treatment. One-way ANOVA followed by post hoc Tukey test; *p*-value indicated on the graph. Data are presented as mean with 95% CI.

To determine whether cromolyn directly affects the viability of the MB49 bladder cancer cell line, we treated these cells directly with 25 μM and 50 μM concentrations of cromolyn (Figure 4a). The results showed that none of the tested concentrations affected MB49 cell numbers (Figure 4b). We extended this investigation to human bladder cancer cell lines, T24 and 5637, treating them with the same concentrations of cromolyn (Figure 4c). Similarly, no changes in cell numbers were observed compared to the control, indicating that cromolyn does not directly affect the viability of human bladder cancer cell lines tested (Figure 4d).

### 2.5. RNA Sequencing Analysis of Established Subcutaneous Tumors from Therapeutic Cromolyn-Treated Mice

**Figure 5:**
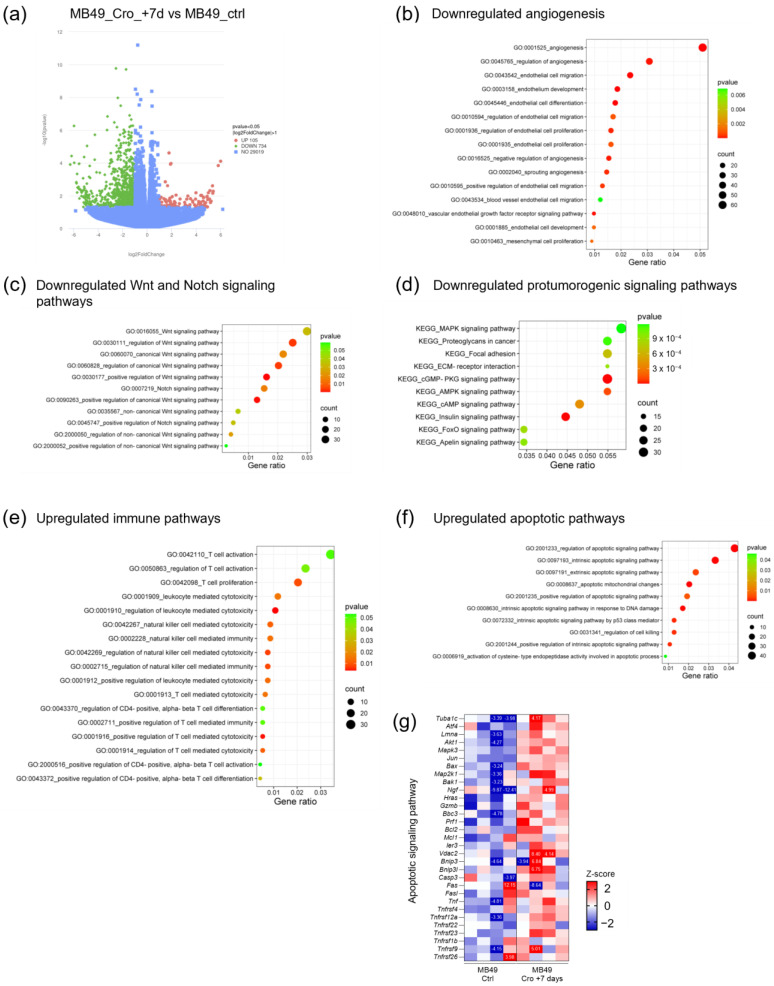
RNA sequencing analysis of established subcutaneous tumors from therapeutic cromolyn-treated mice. (**a**) Volcano plot illustrating differential gene expression in subcutaneous tumors from therapeutic cromolyn-treated mice compared to tumors from control mice. (**b**) The GO biological process enrichment analysis with highlighted downregulated genes associated with angiogenesis. (**c**) The GO biological process enrichment analysis illustrating downregulated genes involved in the Wnt and Notch signaling pathways. (**d**) The KEGG pathway enrichment analysis is presented as a bubble plot, revealing downregulated pro-tumorigenic signaling pathways in subcutaneous tumors from therapeutic cromolyn-treated mice compared to controls. (**e**) The GO biological process enrichment analysis illustrates upregulated genes in immunological pathways following therapeutic cromolyn treatment compared to controls. (**f**) The GO biological process enrichment analysis shows upregulated apoptotic pathways in tumors from therapeutic cromolyn-treated mice compared to controls. (**g**) Heatmap showing the differentially expressed apoptosis-related genes in tumors from therapeutic cromolyn-treated mice compared to controls. Numbers indicated on heatmaps are Z scores out of the legend range.

In the therapeutic cromolyn treatment experiment, RNA sequencing analysis identified 105 upregulated and 734 downregulated genes in tumors from treated mice with a fold change greater than two and *p*-value < 0.05 (Figure 5a). The GO biological process analysis revealed a downregulation of genes related to angiogenesis, specifically the downregulation of the sprouting angiogenesis pathway (Figure 5b). Additionally, genes involved in the Wnt and Notch signaling pathways were downregulated (Figure 5c). The KEGG pathway enrichment analysis further indicated downregulation in several protumorigenic signaling pathways in therapeutic cromolyn-treated mice (Figure 5d). The therapeutic treatment also influenced the immune response, showing a differential effect in comparison to continuous cromolyn treatment. The GO analysis revealed the upregulation of genes related to T-cell response and migration (Figure 5e) accompanied by the upregulation of genes involved in apoptosis (Figure 5f,g). All these results suggest a phenotype of less advanced tumors in mice after therapeutic cromolyn treatment.

### 2.6. Effect of Mast Cell Absence on Subcutaneous MB49 Bladder Tumors

**Figure 6:**
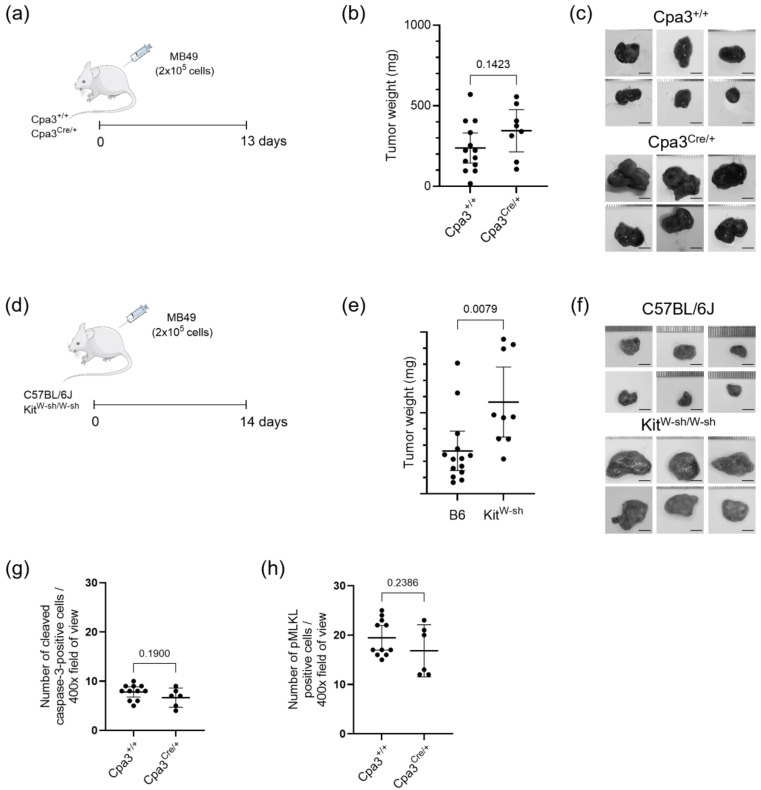
Effect of mast cell deficiency (KO mice) on subcutaneous MB49 bladder tumors. (**a**) Schematic representation of the experimental design for subcutaneous tumor growth utilizing MB49 mouse urothelial carcinoma cell line in MC-deficient mice (Cpa^Cre/+^) and their control (Cpa^+/+^). Scale bar is 5 mm. (**b**) The difference in tumor weight at the end of the experiment in Cpa^Cre/+^ mice compared to control mice is +107.2 mg [95CI − 39.37, +253.57]. *n* = 13 and 8; Student’s *t*-test, *p* = 0.1423. (**c**) Representative images of subcutaneous tumors at the end of the experiment using MC-deficient mice (Cpa^Cre/+^) and their control (Cpa^+/+^). (**d**) Schematic representation of the experimental design for subcutaneous tumor growth utilizing MB49 mouse urothelial carcinoma cell line in MC-deficient mice (Kit^W-sh/W-sh^) and their control (C57BL/6J). (**e**) The difference in tumor weight at the end of the experiment in Kit^W-sh/W-sh^ mice compared to their control is +301.7 mg [95CI + 87.99, 515.4]. *n* = 14 and 9; Student’s *t*-test, *p* = 0.0079. (**f**) Representative images of subcutaneous tumors at the end of the experiment using MC-deficient mice (Kit^W-sh/W-sh^) and their control (C57BL/6J). Scale bar is 5 mm. (**g**) The quantification of cleaved caspase-3-positive cells in immunohistochemically stained subcutaneous tumor sections from Cpa3^Cre/+^ mice and their controls. *n* = 11 and 6; Student’s *t*-test, *p* = 0.1900. (**h**) The quantification of pMLKL^+^ cells in immunohistochemically stained subcutaneous tumor sections from Cpa3^Cre/+^ mice and their controls. *n* = 11 and 6; Student’s *t*-test, *p* = 0.2386. Data are presented as mean with 95% CI.

The stabilization of mast cells was first investigated by using cromolyn in a continuous experiment, targeting mast cells both prior to and during tumor formation. However, to further test tumor development in the complete absence of mast cells and to determine whether cromolyn’s effects are solely due to mast cell stabilization or if other mechanisms are involved, we repeated the syngeneic subcutaneous tumor model in mast cell-deficient mouse strains Cpa3^Cre/+^ and Kit^W-sh/W-sh^ (Figure 6a,d). At the end of the experiment, tumor weight analysis revealed a higher tumor burden in mast cell-deficient Kit^W-sh/W-sh^ mice with a weight difference of +301.7 mg [95CI + 87.99, 515.4], *p* = 0.0079, whereas in mast cell-deficient Cpa3^Cre/+^, there was only a slight increase in tumor burden in comparison to the control, with a weight difference of +107.2 mg [95CI − 39.37, +253.57], *p* = 0.1423 (Figure 6b,c,e,f). Immunohistochemical analysis revealed a reduction in cells positive for activated apoptosis and necroptosis markers, though the differences were not statistically significant (Figure 6g,h).

Altogether, the results presented in this manuscript indicate differential effects of cromolyn depending on the duration and timing of the cromolyn treatment.

## 3. Discussion

The relationship between therapeutic drugs and cancer progression remains a critical area of research, particularly for medications widely used in non-cancer diseases. Cromolyn, a mast cell stabilizer, has the potential for drug repurposing [11]. In this study, we explored the effects of cromolyn beyond allergy management, particularly its influence on the growth and progression of syngeneic subcutaneous bladder cancer models.

By analyzing subcutaneous tumors from continuous and therapeutic cromolyn experiments, we demonstrated that the protocol of treatment determined its effects. In most cromolyn studies, the compound is administered after tumors are already established, similar to our therapeutic cromolyn experiment. These findings align with ours, suggesting that cromolyn exhibits a protective effect. In mice with subcutaneous colon cancer, cromolyn treatment led to reduced tumor weight and volume, though the differences were not statistically significant [14]. In xenograft models of gastric cancer in NOD/SCID mice, the co-administration of cromolyn and mast cells after tumor formation resulted in decreased tumor volumes and slower disease progression [15]. Similarly, Johnson et al. observed that cromolyn administration in mice injected with cells from an extrahepatic biliary cancer cell line slowed tumor growth during the experiment, resulting in smaller tumor sizes midway through the experiment. However, by the end of the experiment, no significant difference in tumor size was observed [16]. Our experiment with the administration of cromolyn after tumor formation also showed a trend in decreased tumor weight and volume, although with *p* = 0.0858. However, a very interesting finding is the contrasting effect of the continuous administration of cromolyn on the subcutaneous bladder cancer model. To our knowledge, no studies have explored the effects of different cromolyn administration protocols within the same tumor model. In addition to this, there are no published studies where cromolyn was administered before the subcutaneous inoculation of cancer cells. An analysis of RNA sequencing data from the continuous cromolyn treatment revealed a strong downregulation of genes involved in immune cell recruitment and their effector functions. The effective activation of the immune system is critical for tumor eradication in its early stages, and its absence may partly explain the observed adverse phenotype [17]. Our analysis also revealed the upregulation of *KRT4* and *KRT15*, markers associated with more dedifferentiated tumors [18,19]. *KRT4*, for example, is highly expressed in the squamous cell carcinoma subtype of urothelial carcinoma, which is linked to poor prognosis [18]. Additionally, we observed the overexpression of *MUC4*, a transmembrane mucin associated with metastatic urothelial carcinoma, and *CLDN4*, a marker linked to more invasive and higher-grade urothelial carcinomas [20,21]. Further analysis highlighted the upregulation of genes involved in the Wnt and Notch signaling pathways, both being critical in developmental processes. These pathways likely contribute to tumor growth and stemness, explaining the observed increase in tumor burden. In bladder cancer xenograft models, Notch inhibition has been shown to reduce tumor mass, while dysregulation of the Wnt/β-catenin pathway can drive the formation of cancer stem cells from early bladder cancer cells and contribute to chemotherapy resistance [21,22,23]. The Gene Ontology (GO) biological processes and KEGG pathway enrichment analysis revealed an inadequate immune response in our continuous cromolyn treatment model. Specifically, we identified the downregulation of key chemokines, such as CCL5, which is essential for recruiting dendritic and NK cells [24]. Additionally, multiple genes involved in T-cell and NK cell responses were also downregulated. Since cytotoxic T-cells and NK cells are critical mediators of cell death, we also examined pathways related to apoptosis and necroptosis [25]. Our findings showed a significant downregulation of genes involved in both apoptotic (*Fas, Bcl2, Casp1, Casp3, Casp6, Casp7, Casp9*) and necroptotic (*Mlkl, Ripk3, IL33*) signaling pathways [26,27]. Immunohistochemical analysis confirmed these findings, showing reduced cleaved caspase-3 and pMLKL-positive cells in tumors from continuous cromolyn-treated mice. Considering the studies that demonstrated that cromolyn affects the viability of different cancer cell lines in vitro, we wanted to determine whether cromolyn directly affects bladder tumor cells [13,14]. Our findings in cromolyn-treated cancer cell lines demonstrated that cromolyn does not directly affect the viability of tumor cells, suggesting that the effect on tumor burden likely occurs through modulation of the tumor microenvironment.

Therapeutic cromolyn treatment presents a distinct outcome compared to continuous treatment. In the continuous experiment, we hypothesized that the treatment influenced the tumor microenvironment from the beginning, shaping its development over time. In contrast, therapeutic treatment targets an already established tumor, along with inducing alterations in the tumor microenvironment as evidenced by the upregulation of gene expression involved in immune response. By this stage, immune cells have already infiltrated the tumor site, and cromolyn appears to enhance the anti-tumor immune response. This is evidenced by the upregulation of genes associated with cytotoxic T-cell and NK cell activity, which play key roles in an effective immune response against the tumor [25]. Additionally, Wnt and Notch, signature pathways in tumor dedifferentiation, were downregulated, while apoptosis pathways were upregulated, aligning with the observed diminished tumor burden. Therapeutic cromolyn treatment also led to a significant downregulation of genes involved in angiogenesis and the formation of new blood vessels. This finding aligns with cromolyn’s role as a mast cell stabilizer, as mast cells are crucial contributors to angiogenesis through the release of various molecules such as VEGF, histamine, and mast cell-specific proteases [28,29]. In support of this finding, previous studies in human urothelial carcinomas have shown a correlation between mast cell density and microvessel density [30].

Considering that cromolyn acts as a mast cell stabilizer, subcutaneous tumors in mast cell-deficient syngeneic mice resulted in a higher tumor burden, similar to the observed effect of continuous cromolyn experiments; however, a less pronounced phenotype was observed. Some studies show that cromolyn actions may extend beyond mast cell stabilization [31,32,33]. In a study investigating the immunomodulatory effects of mast cells in vitro, it was found that cromolyn affects human and mouse mast cells differently [31]. Particularly, the study suggested that cromolyn might also influence other immune cells, such as NK cells and eosinophils, highlighting its potential broader effects on the immune system. This could explain why the subcutaneous tumor experiments in mast cell-deficient mice demonstrated less prominent outcomes compared to the continuous cromolyn experiments. In addition, the difference was more notable in Kit^W-sh/W-sh^ mice compared to Cpa3^Cre/+^ mice, likely due to the comorbidities associated with the Kit^W-sh/W-sh^ strain [34]. Using immunohistochemistry, we stained tumors from mast cell-deficient mice for cleaved caspase-3 and pMLKL, markers of activated apoptosis and necroptosis, respectively. Consistent with our findings from the continuous cromolyn experiments, we observed reduced levels of apoptosis and necroptosis-positive cells, although these differences were not statistically significant.

In summary, our results show that cromolyn exhibits distinct effects on tumor progression and the immune response depending on the timing of its administration. Therapeutic treatment upregulates genes related to cytotoxic T-cell and NK cell activity while suppressing the expression of genes involved in the process of angiogenesis. Conversely, continuous treatment suppresses the expression of genes involved in immune cell recruitment and activation, as well as apoptotic and necroptotic pathways, leading to a higher tumor burden. Given cromolyn’s widespread use in managing allergy symptoms, it is worth investigating its controversial impact on cancer development in humans. On the other hand, future studies need to evaluate cromolyn as a potential candidate for drug repurposing for cancer treatment.

## 4. Materials and Methods

### 4.1. Mice (Animal Work)

C57BL/6J and B6.Cg-Kit^W-sh^HNihrJaeBsmJ mice were ordered from The Jackson Laboratory (Bar Harbor, ME, USA). Cpa3^Cre/+^ mice were received as a gift from Thorsten B. Feyerabend and Hans-Reimer Rodewald, from Division of Cellular Immunology, German Cancer Research Center in Heidelberg, Germany. Only male mice were used in the experiments. All animal experiments followed the guidelines provided by the Animal Health Protection Act and the Instructions for Granting Permits for Animal Experimentation for Scientific Purposes. Ethical approval for the project has been obtained from the Ethics Committee, University of Split Medical Faculty, Croatia, and the Croatian Ministry of Agriculture, Veterinary and Food Safety Directorate, references 003-08/19-03/0003 and UP/I-322-01/19-01/47, respectively. The mice were kept under standard conditions with a 12 h light/dark cycle and were provided with unrestricted access to tap water and food pellets.

### 4.2. Subcutaneous Tumor Experiments

Subcutaneous tumor experiments were conducted using MB49 mice bladder cancer cells. The cells were resuspended in saline and injected subcutaneously to the right flank of 6-to-8-week-old male C57BL/6J, Kit^W-sh/W-sh^, Cpa^+/+^, and Cpa^Cre/+^ mice, weighing approximately 25 g, in the concentration of 2 × 10^5^ cells per 100 µL and with a total volume of 100 µL administered to each mouse.

Daily cromolyn treatment started either 7 days after the initial subcutaneous injection of tumor cells (therapeutic treatment protocol) or one day before the subcutaneous administration of MB49 cells (continuous treatment protocol) until the end of the experiment. In our study designed per protocol, by day 7, the tumors which measured approximately 1 × 1 mm or 2 × 2 mm were evenly distributed between the control and study group, ensuring an equal distribution of animals bearing tumors of similar size. Exclusion criteria were the presence of either non-palpable or overgrown tumors. Cromolyn (HY-B1619, MedChemExpress, Monmouth Junction, NJ, USA) was dissolved in saline at a concentration of 50 mg/kg, and a volume of 100 µL cromolyn solution was administrated intraperitoneally every day for either four days (therapeutic treatment protocol) or 11 days (continuous treatment protocol) until the end of the experiment. The end of the experiment was determined when the tumors approached the maximum diameter of ~15 mm [35]. Mice were sacrificed by cervical dislocation, and subcutaneous tumors were extirpated by a blinded researcher. Exclusion criteria included the presence of ulcers or the intraperitoneal location of the tumor. Tumor weight was measured, and tumors were cut into two parts. Half of the tumor was immediately stored in liquid nitrogen for RNA isolation, while the other half was fixed in 4% paraformaldehyde for paraffin embedding and histological examination.

### 4.3. Immunohistochemistry

Paraffin-embedded tumors were sectioned using a microtome into 4 μm thin slices. Paraffin was removed from the slides, and the sections were rehydrated with water following 100% and 95% ethanol. Sections were boiled for 30 min in 1 mM EDTA buffer (pH 8) for antigen retrieval. Endogenous peroxidases were blocked by incubation with 3% H_2_O_2_. Slides used for anti-cleaved caspase 3 staining were blocked in 1%, while slides used for anti-MLKL were blocked in 15% BSA/TBS-T. Primary antibodies anti-cleaved caspase 3 (Cell Signaling, 9661S, 1:200, Danvers, MA, USA) and anti-pMLKL (Abcam, ab196436, 1:2000, Cambridge, UK) were incubated overnight. After rinsing the primary antibody, sections were incubated for 1.5 h with polyclonal goat anti-rabbit secondary antibody (Dako, P0448, 1:300, Santa Clara, CA, USA). After washing steps, sections were incubated with DAB (Dako, K3468). Hematoxylin was used to counterstain the sections.

In the blinded experiment, immunohistochemically positive cells were counted manually in 3 hot spots under 400× magnification. Analysis of the tumor sections revealed a distinct necrotic core, likely caused by inadequate nutrient and oxygen diffusion, surrounded by actively proliferating tumor tissue. For the quantification of immunohistochemically positive cells, we specifically selected regions of proliferative tumor tissue, ensuring they were sufficiently distant from the necrotic core to maintain reliability. Representative microscopy images were captured using a BX43 microscope (Olympus Corporation, Tokyo, Japan).

### 4.4. RNA Sequencing Analysis

The purification of total RNA from murine subcutaneous tumor tissue was performed using standard Trizol protocol and Minilys tissue disruption. Messenger RNA was isolated from total RNA using poly-T oligo-attached magnetic beads. After fragmentation, the first strand of cDNA was synthesized with random hexamer primers, followed by the synthesis of the second strand using dTTP for a non-directional library. Sequencing was performed with 40 M reads per sample on Illumina Novaseq PE150. Raw sequencing reads were filtered for low-quality reads or reads with adapters and aligned using HISAT2 to the mm10 reference genome. Differential gene expression analysis was performed using DESeq2 [36], and genes with log2FC ≥ ±1 were considered differentially expressed with *p*-value ≤ 0.05.

Differential expression analysis, as well as GO enrichment and KEGG enrichment analyses, were analyzed on the cloud platform NovoMagic (https://ocsseurope.novogene.com/oauth/login, accessed on 1 October 2024). Gene set enrichment analysis (GSEA) was additionally conducted using the GenePattern GSEA module on DESeq2 normalized data (version 20.4.0) on MSigDB gene set collections with 1000 permutations per gene set performed [37]. Enrichment dot bubble plots were generated using tools available on the online platform Bioinformatics (https://www.bioinformatics.com.cn, accessed on 1 October 2024). Z-score heat maps were generated using FPKM-normalized RNA sequencing data and plotted using GraphPad Prism 10 software.

### 4.5. Cromolyn Treatment on Bladder Cancer Cell Line

Mouse bladder cell line MB49 was a gift from Prof. Thomas Tötterman (Uppsala University, Uppsala, Sweden) and human bladder cancer cell lines T24 (ACC 376) and 5637 (ACC 35) were purchased from DSMZ (Braunschweig, Germany). DSMZ ensures the authentic origin of the cells, while we independently verified the identity of MB49 cells through karyotyping and an assessment of their specific morphology. Additionally, all cell lines were tested monthly for mycoplasma contamination. The cells were cultured in DMEM, and once they reached 70% confluence, cells were treated for 48 h with 25 μM and 50 μM concentrations of cromolyn dissolved in DMSO. The control group was treated only with DMSO. At the end of treatment, cells were trypsinized, and the total number of cells per well counted.

### 4.6. Statistics

All statistics were performed using GraphPad Prism 10 software. The study was designed as an exploratory study; thus, effect size indicators include mean values with a 95% CI. The effect of cromolyn on bladder cancer was tested with a two-tailed Student’s t-test, which was used to analyze differences in tumor mass and differences in the number of positive cells after immunohistochemical staining. A one-way ANOVA followed by a post hoc Tukey test was used to analyze differences in the number of MB49, T24, and 5637 cells after cromolyn treatment. Individual statistical tests were indicated for each experiment in the figure legend. All graphs illustrating differences in tumor mass and the number of positively stained cells display the means along with their corresponding confidence interval (95CI).

## Data Availability

The data in this study are available from the corresponding author upon request.
